# Vocal cord paralysis secondary to spontaneous internal carotid dissection: case report and systematic review of the literature

**DOI:** 10.1186/1916-0216-42-34

**Published:** 2013-05-13

**Authors:** TT Jean Nguyen, Han Zhang, Peter T Dziegielewski, Robert Seemann

**Affiliations:** 1 College of Medicine; Université de Sherbrooke, Sherbrooke, QC, Canada; 2 Division of Otolaryngology-Head and Neck Surgery, University of Alberta, Edmonton, AB, Canada

## Abstract

**Objectives:**

To present a rare case of unilateral vocal cord paralysis (VCP) secondary to spontaneous internal carotid artery dissection and to perform a literature review.

**Case report:**

A 35-year-old male presented to the emergency department with acute onset hoarseness and dysphagia. History, physical exam and laryngoscopy revealed left sided VCP without obvious cause. Magnetic Resonance Imaging (MRI) demonstrated a left internal carotid artery dissection of unknown etiology. Neurovascular surgery was consulted and treatment with aspirin was initiated. The dysphagia and hoarseness resolved in 12 weeks with long-term neurosurgery follow-up as the management plan.

**Methods:**

Systematic literature review was conducted by 3 independent reviewers. Since 1988 only 9 cases of VCP due to internal carotid artery dissection have been reported. These were reviewed for: demographics, diagnostic method, treatment and vocal cord function.

**Results:**

7 patients had unilateral while 2 had bilateral VCP. MRI was used for diagnosis in 7 cases and 5 cases utilized a type of angiography. All received antithrombotic treatment with 5 out of the 9 patients experiencing vocal cord recovery in an average of 7.2 weeks.

**Conclusion:**

MRI is crucial in the work-up of idiopathic VCP. If an ipsilateral internal carotid artery dissection is found, antithrombotic treatment is initiated with an expectation that vocal cord mobility is likely to return.

## Background

Spontaneous internal carotid artery dissection is a rare cause of lower cranial nerve palsy, which often leads to vocal cord paralysis (VCP). More common causes include infiltrating skull base tumours, trauma or inflammatory disease [1]. The incidence of internal carotid artery dissection ranges from 2.5 to 3 per 100 000 [2] affecting mostly men in their fifties [3]. The first reported case of spontaneous ICA was in 1959 [4]. Since then, few cases associated with lower cranial nerve palsies have been described.

In this article, a VCP secondary to an internal carotid artery dissection is described and a systematic review of the literature is performed. The objective of this report is to delineate optimal diagnostic and treatment modalities and describe the expected prognosis.

## Methods

Health research ethics board approval at the University of Alberta was obtained. All 17 Otolaryngology-Head and Neck Surgery attending physicians at the University of Alberta were surveyed to identify any known cases of vocal cord paralysis following carotid dissection. From 1998–2011 one case was identified. The outpatient and inpatient medical records for this patient were obtained and reviewed for: age, gender, presentation, work-up, treatment and functional outcomes.

A systematic review of the literature was conducted using Pubmed, Medline, and Scopus. The search terms “vocal cord paralysis”, “vagus/vagal nerve palsy”, “dysarthria” or “dysphagia” were each combined with “carotid dissection” or “carotid artery dissection”. As such 8 search strategies were formed. 3 independent reviewers employed these strategies to identify all known cases of vocal cord paralysis following carotid dissection in the English literature. All relevant abstracts were reviewed and potentially pertinent articles were thoroughly examined. Any relevant papers referenced in these articles were also reviewed.

## Case presentation

A 35 year old male presented to the emergency room at the University of Alberta Hospital with a three week history of progressive dysphagia and hoarseness. The patient denied any other upper aerodigestive tract symptoms. He had a long term history of epilepsy but was symptom free for ten years after long term treatment with valproic acid. There was no history of trauma or surgery. Complete head and neck examination including flexible nasopharyngoscopy was unremarkable except for a paralyzed left vocal cord in the paramedian position. CBC-D, electrolytes, urea, creatinine, TSH, CK, ALT, ALP, ESR, CRP, C- and P-ANCA were all found to be within normal range. Computerized tomography scanning (CT) of the neck was also performed; however, no pathology to account for the VCP was identified. Thus, the patient was diagnosed with idiopathic VCP.

**Figure 1:**
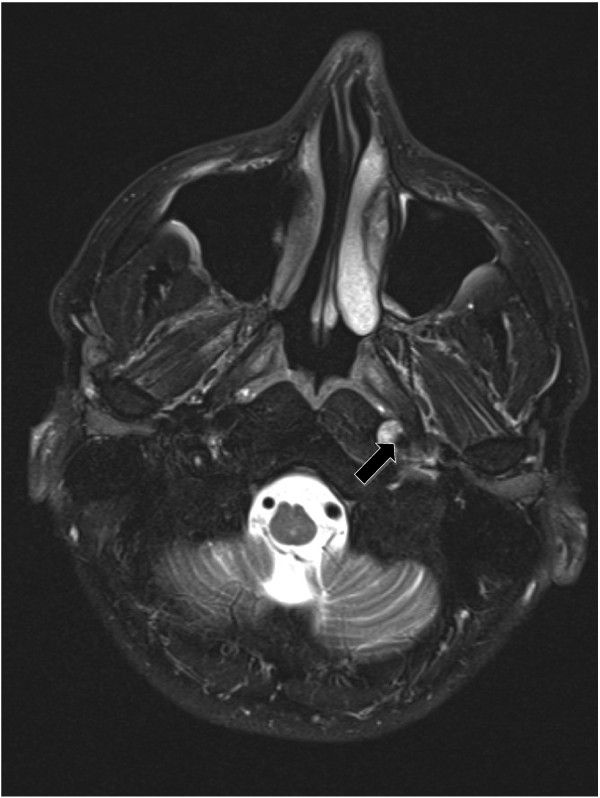
T2-weighted MRI showing a left sided non-occlusive dissection extending to the level of the petrous segment (arrow).

**Figure 2:**
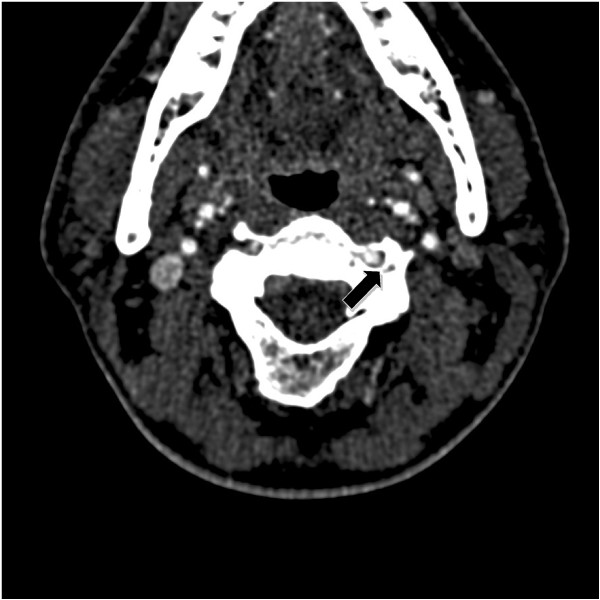
CT angiography showing a left vertebral artery dissection located at the C4 and C5 transverse foramina (arrow).

Otolaryngologic follow-up one week later showed persistence of the VCP, which warranted further imaging. A magnetic resonance imaging scan (MRI) of the brain, neck and chest was performed to trace the path of the vagus and recurrent laryngeal nerves bilaterally as well as to rule out the possibility of multiple sclerosis or amyotrophic lateral sclerosis. The detailed images revealed a non-occlusive dissection of the cervical portion of the left internal carotid artery, extending into the petrous segment (Figure 1). Additionally, CT angiography showed a left vertebral artery dissection at the C4 and C5 transverse foramina (Figure 2) along with a saccular-like aneurysm arising from the distal cervical internal carotid artery.

The patient was then referred to a neurovascular surgeon for the internal carotid artery dissection and a speech language pathologist for his dysphagia. He was started on 81mg of aspirin daily and within 3 weeks his dysphagia and dysphonia had resolved. At 3 months follow-up he was found to have bilateral full vocal cord mobility. At the recommendation of the neurovascular surgeon he will be treated with long term low dose aspirin and close follow-up.

## Results

Literature review identified 32 potential cases within 32 papers since 1988. Of those, 9 cases of VCP secondary to carotid dissection were found in 9 papers. Table 1 demonstrates the case specifics. The average age of the patients was 47.1 years with all the patients being male. All of the patients had at least a unilateral carotid artery dissection, while 2 patients had a bilateral carotid artery dissection.

**Table 1:** Cases of vocal cord paralysis secondary to spontaneous carotid dissection

**Articles**	**Author**	**Age**	**Sex**	**Side of CAD**	**Side of VCP**	**Diagnosis**	**Treatment**	**Time to recovery of vocal cord function**
**Vocal Cord Paralysis due to Spontaneous Internal Carotid Dissection**	Nguyen, T. [2011]	35	M	Left	Left	CTA	ASA	12 weeks
						MRI		
**Tenth and Twelfth Nerve Palsies in a Patient with Internal Carotid Artery Dissection Mistaken for Cervical Mass Lesion**	Arnoldner, C. [2010]	52	M	Left	Left	CTA	Phen-procoumon	4 weeks
						MRI		
**Unusual Manifestations of Bilateral Carotid Artery Dissection: Dysphagia and Hoarseness**	Isildak, H. [2010]	40	M	Bilate-ral	Left	CTA	Anticoagulants	N/A
						MRI	(Not specified)	
**A carotid artery dissection presenting with dysphagia due to a dilation of upper oesophagus**	Vaes, M. [2007]	57	M	Left	Left	MRA	Heparin	N/A
						Doppler U/S	Comuadin	
**“Spontaneous” Internal Carotid Artery Dissection Following Cough Induced by ACE Inhibitor Therapy**	Simionescu [2004]	64	M	Right	Right	Catheter cerebral angiography	ASA	12 weeks
**Vocal Cord Palsy Resulting From Spontaneous Carotid Dissection**	Wessels, T. [2003]	43	M	Right	Right	MRI	Heparin IV	N/A
							Warfarin	
**Spontaneous internal carotid artery dissection with isolated vagus nerve deficit**	Moussouttas, M. [1998]	40	M	Right	Right	MRI	N/A	N/A
**Isolated Vagal Nerve Palsy Associated with a Dissection of the Extracranial Internal Carotid Artery**	Nusbaum, A. [1998]	40	M	Right	Right	MRI	Heparin IV	6 weeks
							Warfarin	
**Lower Cranial Nerve Palsies Due to Internal Carotid Dissection**	Waespe, W. [1988]	41	M	Bilateral	Left	MRI	Surgical aneurysm obliteration	2 weeks

Abbreviations: M, male; CAD, carotid artery dissection; VCP, vocal cord paralysis; MRA; Magnetic resonance angiography; MRI, Magnetic resonance imaging; CT scan, computed tomography scan; CTA, computed tomography angiography; U/S, Ultrasound; Dx, Method of diagnosis; Tx, Treatment; N/A, not available.

The diagnosis of internal carotid artery dissection was made using MRI in all of the cases. CT angiography was used as part of the diagnosis in 3 of the 9 cases. All patients were treated with antithrombotic therapy except for the cases described by Waespe [5] and Moussoutas [6]. Surgical aneurysm obliteration was the treatment of choice in Waespe’s case due to progressive symptoms that failed to respond to medical therapy after one month. 5 of the 9 cases reported resolution of the vocal cord paralysis with an average recovery time of 7.2 weeks.

## Discussion

VCP is a rare manifestation of ICA dissection and is found in 16% of cases [7]. The most common etiologies of VCP are extralaryngeal tumours, surgical insult, and external trauma [8]. ICA dissections are usually secondary to trauma and congenital connective tissue defects. Cystic medial necrosis, fibromuscular dysplasia, type IV Ehlers-Danlos syndrome, Marfan syndrome, ICA redundancy, idiopathic regressing arteriopathy, hypertension, and α1-antitrypsin deficiency have also been found to be causes of ICA dissection [3,9-11]. The average age of onset in these patients is 45 years old and it more commonly affects men in a ratio of 3:2 [12]. Typically patients present with pain, headache, Horner’s syndrome, lower cranial nerve palsies, and cerebral or ocular transient ischemic attacks or strokes [13]. The proximity of the cranial nerve to the ICA is usually the cause of the symptoms via mass effect [8]. While initial presentation is often fairly benign, embolic strokes [14], cervical aneurysm [15] and transient ischemic attacks [16] are complications that may occur.

MRI is typically the initial modality used to diagnose the dissection, while angiography is the imaging method used to better characterize and localize the pathology [15]. On CT scanning; large ICA dissections may be heralded by areas of hypodensity. MRI typically shows findings of hyperintensity on T1 and T2 signals or an outline of an intramural hematoma [15]. Medical management, specifically in the form of antithrombotic treatment using aspirin, or warfarin is the treatment of choice for ICA dissections [17]. The rationale for treatment is that infarcts secondary to ICA dissections are thromboembolic rather than hemodynamic. While anti-thrombotic therapy doesn’t offer resolution of the dissection, it does allow for a high rate of recanalization of the artery within the first 2–3 months and for the prevention of ischemic complications [17].

Definitive ICA dissection treatments include balloon angioplasty, distal thrombectomy, ligation of the distal carotid artery, and external – internal carotid bypass [18,19]. These options are typically recommended for patients who present with recurrent ischemic symptoms and who are not responsive to conservative therapy [20]. However, such invasive measures can be complicated by the development of cerebral ischemia, intracranial aneurysms or cranial neuropathies [19,21,22]. Long-term follow-up is often warranted in these patients and is best left to a neurovascular surgeon.

Prognosis of ICA dissection is encouraging. In 23% to 85% of the cases, recovery is either fully or nearly fully attained [21,22]. Mokri compared different treatments for ICA dissection on 36 patients regrouping antiplatelet therapy (ASA or ASA with dipyridamole), anticoagulant therapy, superficial temporal-middle cerebral artery bypass and no treatment. Antiplatelet therapy for those patients lasted 3–5 months. Close follow-up for 34 months revealed that no patients experienced recurrent ischemic symptoms after cessation of the antiplatelet medication. All patients except 2 had complete resolution of their symptoms which coincides with our own experience in terms of treatment and recovery.

## Conclusion

Although rare, ICA dissection should be considered in the differential diagnosis of potentially idiopathic VCP. Diagnosis is best made with MRI and the extent of dissection is assessed with CT angiography. Patients without recurrent ischemia are best managed with conservative anticoagulation, which often needs be no more than a daily low dose of aspirin. Long term follow-up suggests that VCP after ICA dissection recovers on average within 7.2 weeks.

## Ethics approval

This study was approved by the University of Alberta’s Health Research Ethics Board.

## Consent

Written informed consent was obtained from the patient for publication of this report and any accompanying images.

NB: The corresponding author has full access to the data in this study and takes responsibility for the integrity of the data and accuracy of the data analysis.

## Competing interests

The authors’ declared that they have no competing interests.

## Authors’ contributions

TN was responsible for study design, obtaining the figures, and drafting the manuscript. HZ was responsible for study design, data calculation, and drafting the manuscript. PD was responsible for study design and drafting the manuscript. RS was the principal investigator for this study. All authors read and approved the final manuscript.

## Presentation

This study was presented by T.T. Jean Nguyen at the 66th *Canadian Society of Otolaryngology-Head and Neck Surgery* annual meeting in Toronto, ON on May 20–22, 2012.

## Submission

This material has never been published and is not currently under evaluation in any other peer-reviewed publication.
